# Single Molecule Thermodynamic Penalties Applied to Enzymes by Whispering Gallery Mode Biosensors

**DOI:** 10.1002/advs.202403195

**Published:** 2024-07-12

**Authors:** Matthew C. Houghton, Nikita A. Toropov, Deshui Yu, Stefan Bagby, Frank Vollmer

**Affiliations:** ^1^ Living Systems Institute University of Exeter Exeter Devon EX4 4QD UK; ^2^ Department of Physics and Astronomy University of Exeter Exeter Devon EX4 4QD UK; ^3^ Department of Life Sciences University of Bath Bath Somerset BA2 7AY UK; ^4^ Optoelectronics Research Centre University of Southampton Southampton SO17 1BJ UK; ^5^ National Time Service Centre Chinese Academy of Sciences Xi'an 710600 China

**Keywords:** biosensors, enzymes, optical forces, optoplasmonics, peptides and proteins, plasmonics

## Abstract

Optical microcavities, particularly whispering gallery mode (WGM) microcavities enhanced by plasmonic nanorods, are emerging as powerful platforms for single‐molecule sensing. However, the impact of optical forces from the plasmonic near field on analyte molecules is inadequately understood. Using a standard optoplasmonic WGM single‐molecule sensor to monitor two enzymes, both of which undergo an open‐to‐closed‐to‐open conformational transition, the work done on an enzyme by the WGM sensor as atoms of the enzyme move through the electric field gradient of the plasmonic hotspot during conformational change has been quantified. As the work done by the sensor on analyte enzymes can be modulated by varying WGM intensity, the WGM microcavity system can be used to apply free energy penalties to regulate enzyme activity at the single‐molecule level. The findings advance the understanding of optical forces in WGM single‐molecule sensing, potentially leading to the capability to precisely manipulate enzyme activity at the single‐molecule level through tailored optical modulation.

## Graphical abstract

Turnover of enzymes bound to optoplasmonic sensors can be detected at the single‐molecule level. By modulating the strength of the electric field generated by the plasmonic gold nanoparticle, optoplasmonic sensors may be capable of generating forces that oppose turnover and that could therefore be used to control enzymes at the single molecule level. Created with BioRender.com

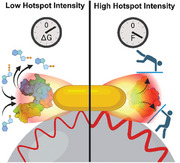

## 1 Introduction

Atomic resolution protein structure studies have been facilitated in recent years by advances in tools such as X‐ray crystallography, nuclear magnetic resonance, cryo‐electron microscopy, and structure prediction algorithms, leading to better fundamental understanding and, for example, improvements in drug design.^[^ ^1^ ^]^ Our understanding of protein motion and of protein manipulation, however, is less well‐developed. Expensive equipment and intensive simulations have allowed visualisation of protein motions over a few µs but larger‐scale domain motions relevant to catalysis typically occur over longer timescales.^[^ ^2^ ^]^ Novel methods of analysis are required to understand the thermodynamics of these larger scale motions, as well as how we can manipulate these motions to control enzyme activity. Understanding the mechanical forces involved in these conformational changes, moreover, could lead to improved design of enzymes for biomedical and biotechnological applications.

Whispering gallery mode (WGM) sensors represent an exciting and expanding research area with several remarkable biosensing applications, including single nanoparticle detection,^[^ ^3^, ^4^ ^]^ monitoring DNA hybridization,^[^ ^5^ ^]^ and intracellular microlaser sensing.^[^ ^6^, ^7^ ^]^ WGM sensors are typical WGM resonators, which have unsurpassed quality (Q) factors values reaching ≈10^10[^ ^8^ ^]^ and narrow resonance lines, making them sensitive to minuscule perturbations of the surrounding media. Spherical WGM microresonators provide high sensitivity for label‐free detection of numerous analytes but their relatively large size (typical diameter of 100 µm) often limits their use for single molecule detection.^[^ ^9^ ^]^ Optoplasmonic signal enhancement through attachment of gold nanorods to the microresonator is a popular method to effectively reduce the mode volume, allowing detection of even small molecules at the nanorod tips.^[^ ^10^ ^]^ Biochemical applications of this technique are emerging, as illustrated by detection of DNA polymerase conformational change^[^ ^11^ ^]^ and sensing of activation heat capacity.^[^ ^12^ ^]^ The former investigation^[^ ^11^ ^]^ involved the first detection of enzyme turnover events by a plasmonically enhanced WGM (PE‐WGM) sensor, outlining an understanding of how a change in enzyme polarisability or volume will result in a change in WGM resonance wavelength. Subramanian et al. (2021) provided evidence for the macromolecular rate theory of enzyme catalysis by measuring negative heat capacity change ($\Delta C_{p}^{‡}$) via MalL temperature‐dependent single‐molecule kinetics.^[^ ^12^ ^]^ Modulation of the near‐field gradient of the plasmonic nanostructures is expected to affect enzyme turnover,^[^ ^7^ ^]^ but this has not been demonstrated experimentally.

**Figure 1:**
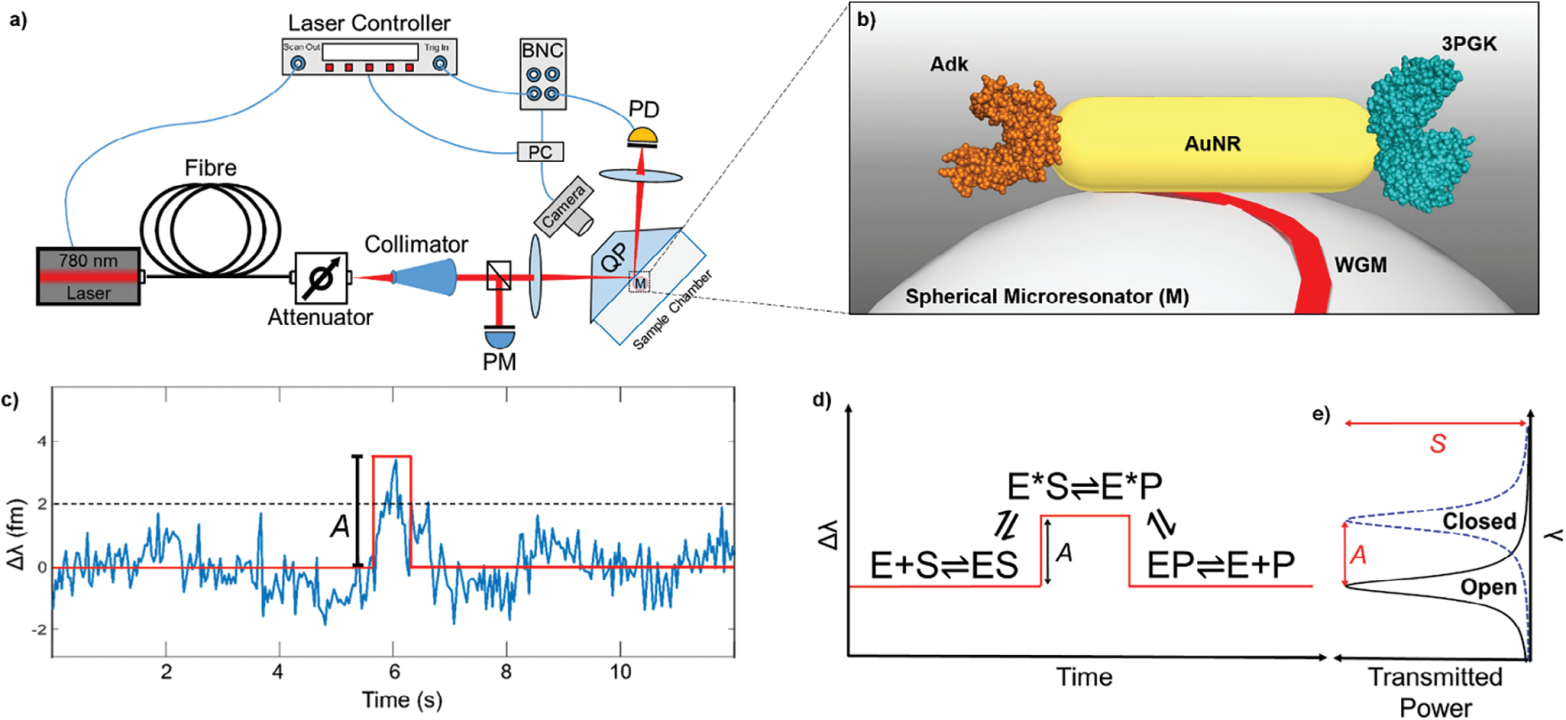
Optoplasmonic sensing of enzyme conformational change. a) Configuration of whispering gallery mode (WGM) sensor as a force gauge (dynamometer). A 780 nm laser provides a scanning continuous wave to a quartz prism (QP), focussed via a collimator and optics, where a spherical microresonator (the microcavity, M) is coupled via the evanescent field formed. The spectrum produced is detected at the photodetector (PD), with power set via an attenuator, and monitored with a power meter (PM). b) Nanoscale depiction of a plasmonic enhancer bound to the silica microresonator. The plasmonic gold nanorods (AuNR) are excited by a WGM, forming a near‐field enhancement: the plasmonic hotspot. Within this hotspot is a single enzyme molecule, either Adk or 3PGK. c) WGM mode wavelength change (Δλ) during 3PGK turnover, monitored by PE‐WGM at *I* ≈ 48 MW cm^−2^, following addition of 3PGA and ATP (blue). The immobilised 3PGK will bind the substrates and undergo large scale conformational changes during catalysis, causing spiked signals of amplitude, *A*. Signals are modelled as a rectangular profile (red), represented by Equation (3) below, where signals are identified at a significance level of three times the standard deviation (Δλ > 3σ). d) Rationale for accessing intrinsic parameters from WGM data. An immobilized enzyme (E) on the surface of an AuNR will bind substrates (S) from solution, forming the open enzyme‐substrate complex (ES). The enzyme then undergoes conformational change, causing a polarisability change that results in Δλ shifts of amplitude *A*, forming the closed enzyme‐substrate complex (E*S). The catalytically competent E*S converts substrates to products to form the closed enzyme‐product complex (E*P). The length of the signal is represented by τ. The E*P complex undergoes another conformational change, opening to allow product (P) release via the open enzyme‐product complex (EP). This creates rectangular profiles, represented by Equation (3) below.^[^ ^12^ ^]^ e) The transmission output from the WGM sensor. A Lorentzian curve of full‐width‐at‐half‐maximum (δλ) and coupling percentage (*S*) forms where photons are coupled to the microresonator.

We report a novel single molecule *dynamometer* (force gauge) combining optical tweezer, plasmonic, and WGM biosensor technologies (**Figure** **1**). We have developed a quantitative analysis of WGM spectra, quantifying the spectral shifts of WGM resonances recorded during enzyme turnover alongside the optical power coupled to the microcavity and resulting WGM intensity at the enzyme. This permits calculation of the work done on the enzyme and of Gibbs free energy penalties imposed on conformational change by the sensor, which has not previously been demonstrated for enzymes coupled to optoplasmonic sensors. We also introduce an optoplasmonic force equation that allows calculation and comparison of optical forces acting on different enzymes. For comparison, we use 3‐phosphoglycerate kinase (3PGK) and adenylate kinase (Adk) which both exhibit hinge‐bending conformational changes.

## 2 Results

**Figure 2:**
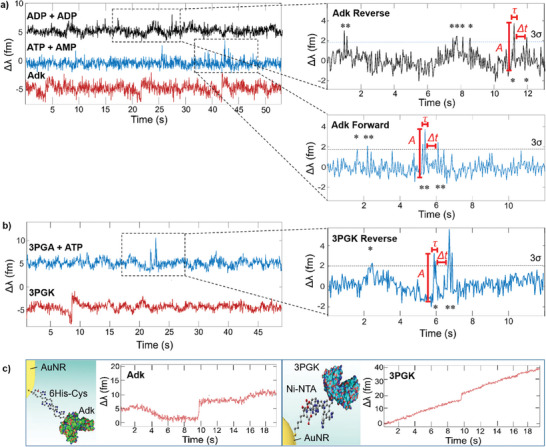
Observation of turnover signals during optoplasmonic sensing of 3PGK and Adk. a) Detrended WGM mode wavelength changes (Δλ) during Adk binding (red) and turnover in both the forward (blue) and reverse (black) reaction directions. A 3 fm shift is observed when Adk forms gold‐thiol bonds – three separate binding events can be seen here. No turnover signals can be observed until substrates (ATP + AMP for forward reaction, ADP + ADP for reverse reaction) are added to the chamber where Adk binds the substrates and undergoes large scale conformational changes during catalysis. Signals (*) are seen as single‐peaked spikes of 3–4 fm in amplitude (*A*), at Δλ > 3σ significance level, length τ, and separated by dwell time Δ*t*. b) Detrended Δλ during 3PGK binding (red) and turnover, following addition of 3PGA and ATP (blue). A 5 fm red shift in mode λ occurs when 3PGK binds in the near field of the plasmonic AuNR. The immobilised 3PGK will bind the substrates and undergo large scale conformational changes during catalysis, hence causing spiked signals. c) Raw Δλ data of Adk binding via Cys‐gold interactions and of 3PGK binding via Ni‐His interactions.

**Figure 3:**
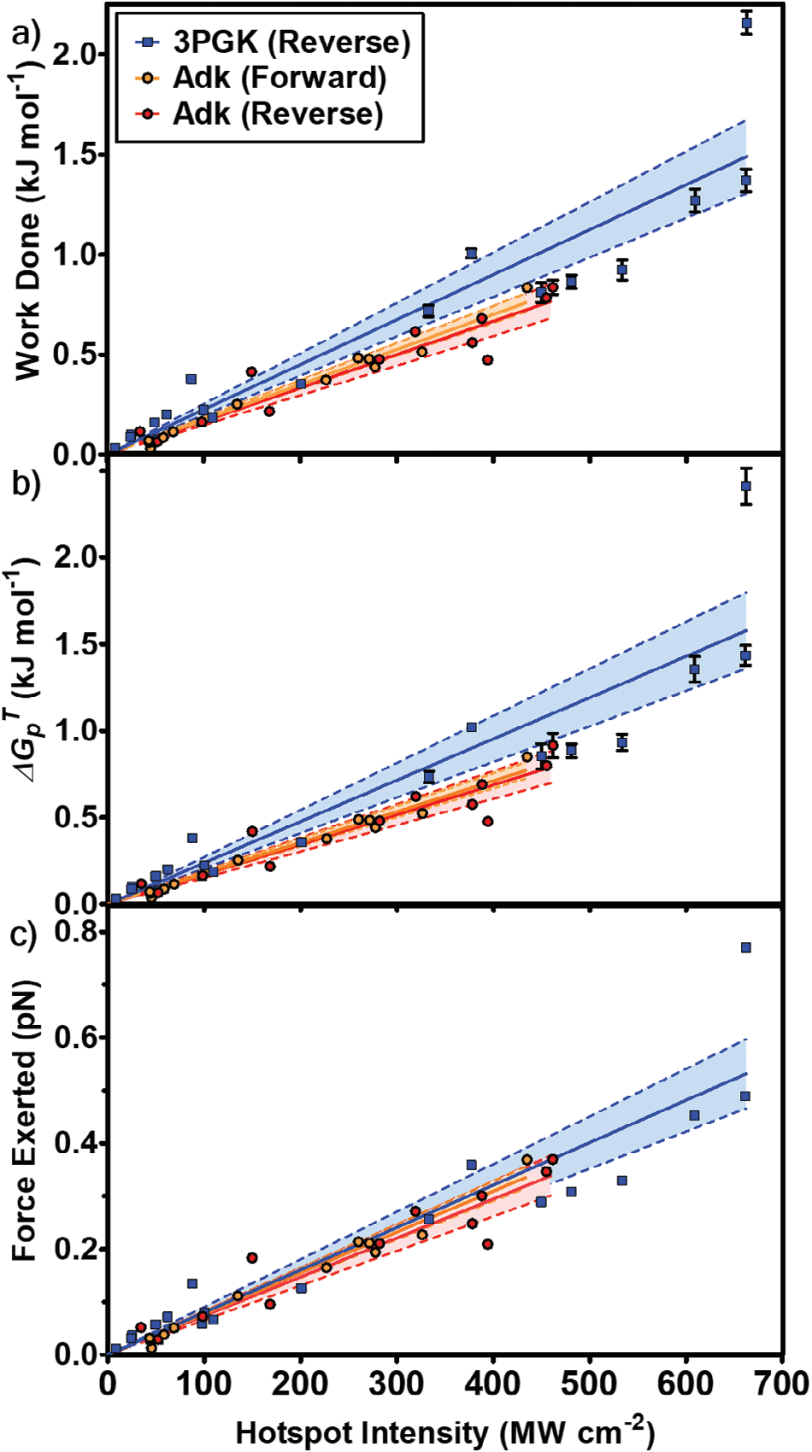
Thermodynamic quantities and forces extracted from optoplasmonic sensing. a) Work done by the sensor (*w*) measured during optoplasmonic biosensing experiments at different WGM intensities at the enzyme (*I*) with standard error bars. A linear relationship is observed between *w* and *I* for 3PGK (reverse – blue), Adk (forward – orange) and Adk (reverse – red). Work done on 3PGK is greater than the work done on Adk. 95% confidence intervals are represented by corresponding shaded regions bound by dotted lines. b) Free energy penalty ($\Delta G_{p}^{T}$) measured from PE‐WGM, utilising Jarzynski's estimator (Equations (13), (14), (15)). c) Apparent force exerted by the PE‐WGM sensor on 3PGK and Adk using $F=\frac{w}{\bar{d_{E}}}$, where *F* is the apparent force and $\bar{d_{E}}$ is the average enzyme diameter. Controlling for enzyme diameter shows that the same force, dependent on *I*, is being applied to each enzyme.

3PGK and Adk were bound separately to plasmonic gold nanorods (AuNR) coupled to a microsphere supporting WGMs (Figure S1, Supporting Information), aided by preferential binding at the nanorod tips.^[^ ^13^, ^14^, ^15^, ^16^ ^]^ In the presence of substrates, 3PGK and Adk undergo an open‐to‐closed‐to‐open conformational transition involving rearrangements of atoms (Table S1, Supporting Information). Polarisability changes ensue as parts of the enzyme move along optical field gradients during turnover (**Figure** **2**).^[^ ^17^, ^18^, ^19^, ^20^ ^]^ This results in work being done on the enzyme by the sensor as the atoms move through the electric field of the plasmonic hotspot.^[^ ^7^ ^]^ We establish and quantify this work done by the sensor during enzyme turnover when atoms of the enzyme move along the near‐field gradients at different WGM intensities. The average work done (*w*) was measured as 0.03‐2.16 and 0.03‐0.84 kJ mol^−1^ for 3PGK and Adk, respectively, increasing proportionally with increase in WGM intensity at the enzyme (*I*) (Tables S2 and S3, Supporting Information). The proportionality constant Δ*w*/Δ*I* was found to be 2.25 J cm^2^ MW^−1^ mol^−1^ for 3PGK and 1.69 J cm^2^ MW^−1^ mol^−1^ for Adk (**Figure** **3**a).

To understand the work done by the sensor on the enzyme as a thermodynamic penalty applied to enzyme conformational change, *w* was converted to Gibbs free energy change (Δ*G*) using Jarzynski's estimator,^[^ ^21^, ^22^, ^23^ ^]^ giving values of 0.03‐2.41 kJ mol^−1^ for 3PGK and 0.07–0.92 kJ mol^−1^ for Adk (Figure 2b). These differ from known values for the free energy of open‐to‐closed transition (Δ*G_c_*), which are 4–38 kJ mol^−1^ for kinases^[^ ^24^, ^25^, ^26^ ^]^ and other transferases,^[^ ^27^, ^28^ ^]^ with values for 3PGK ≈4 kJ mol^−1[^ ^25^ ^]^ and Adk ≈ 33.5 kJ mol^−1^.^[^ ^29^ ^]^ Notwithstanding potential limitations of Jarzynski's estimator,^[^ ^30^, ^31^, ^32^ ^]^ the PE‐WGM‐measured Δ*G* values here are smaller than Δ*G_C_* and therefore are not those of Δ*G_C_* but defined herein as a thermodynamic penalty ($\Delta G_{p}^{T}$) that the enzyme must overcome when placed in the near field of an excited plasmonic nanorod in order to undergo the open‐to‐closed transition for turnover to take place.^[^ ^7^ ^]^

In the following we present quantitative analysis focussed on accessing the dynamics of single molecules by coupling them with the cavity of a whispering‐gallery mode (WGM) sensor, as presented above for 3PGK and Adk.^[^ ^12^, ^33^, ^34^, ^35^ ^]^ In such an arrangement, a molecule undergoing transitions between different conformational states causes a series of shifts Δλ  =  (Δλ_1_, Δλ_2_, . . .) to the field wavelength at times *t*  =  (*t*_1_,*t*_2_, . . .). The associated linewidths δλ  =  (δλ_1_, δλ_2_, . . .) are also measured. Below, we use the notation Δ*X* :   =  *X_i_*  −  *X*_0_ *and* *X_i_* :   =  *X*(*t*), where *X* denotes a generic variable.

Using perturbation theory to analyse the Δλ in terms of permittivity change:^[^ ^36^, ^37^ ^]^

(1) $$\frac{\Delta \lambda }{\lambda_{0}}=\frac{\alpha_{ex,i}}{2V_{eff,i}}$$

where

(2) $$V_{eff,i}=\frac{\int \varepsilon \left(r\right){\left|E\left(r\right)\right|}^{2}dr}{\Lambda \varepsilon \left(r_{s,i}\right){\left|E_{max}\left(r_{s,i}\right)\right|}^{2}}\;$$

*V*_*eff*,*i* –_ mode volume, *E*  − mode field, ε – dielectric permittivity, λ_0_ – the initial wavelength, *r*_*s*,*i*_ – the scatterer's position, α_*ex*,*i*_ – excess polarisability caused by the change to the sensor environment as each molecule moves, and Λ  − the local‐intensity enhancement factor at position r_0_ when accounting for plasmonic enhancement by AuNRs, the value of which in this work is roughly 800. E(r) can be calculated from formulae described in Balac (2019).^[^ ^38^ ^]^

Upon coupling enzyme molecules to the PE‐WGM sensor, substrates are added. This generates a series of step‐like events (Figures 1 and 2); we use a rectangular profile to model each of these (Figure 1d):

(3) $$\Delta \lambda \left(t_{i}\right)\approx A\;\mathrm{rect}\left(\frac{t_{i}-\omega }{\tau }\right)$$

where the amplitude *A*, the width τ and the time position ω are free parameters. We identify the work done (in J mol^−1^) by the sensor at time *t* as

(4) $$w_{i}=N_{A}\Delta E_{i}=N_{A}N_{\mathrm{in}}h\Delta v_{i}$$

In the first equality, *N_A_* is Avogadro's number and Δ*E_i_* is the difference in energy between two conformational states: one at energy *E*_0_—tentatively, an equilibrium state—and a second state at energy *E_i_*. In the second equality, *N_in_* is the intracavity photon number, *h* is Planck's constant, and Δν_*i*_ is the associated frequency shift. We rewrite Equation (4) in terms of the parameters that we can control below.

Using ν_0_ =  *c*/λ_0 _and Δλ_*i*_ ≪ λ_0_, where c is the speed of light, we have that Δν_*i*_/ν_0_ ≈ −Δλ_*i*_/λ_0_. Hence, 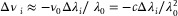, which in turn allows us to rewrite Equation (4) as:

(5) $$w_{i}\approx \;-\;\frac{N_{A}N_{in}hc\Delta \lambda_{i}}{\lambda_{0}^{2}}$$

Next, we estimate the intracavity photon number *N_in_* by using *N_in_* = *a_i_* *N_i_*, where *N_i_*  =  4*P*/(κ*h*ν_0_)  =  4*P*λ_0_/(κ*hc*) is the number of photons reaching the cavity and *a_i_* is defined as *a_i_* = κ_*in*_ /κ.^[^ ^39^, ^40^, ^41^ ^]^ Here, *P* is the input‐light power, κ is the total loss rate of the cavity, and κ_*in*_ is the fiber‐cavity coupling rate, so that *a* is dimensionless. As a consequence, Equation (5) can be expressed as:

(6) $$w_{i}\approx -\frac{4N_{A}a_{i}P\Delta \lambda_{i}}{\kappa \lambda_{0}}$$

We now focus on *a_i_* and κ. The former is found by solving $a_{i}^{2}-a_{i}+\left(S_{i}/4\right)=0$, where *S_i_* is a (dimensionless) coupling efficiency. Assuming that we are in the under‐coupled regime, this leads to $\;a_{i}=\left(1\;-\;\sqrt{1\;-\;S_{i}}\right)/2$. As for κ, this can be expressed in terms of the WGM linewidth δλ_*i*_ – measured in units of length – as $\kappa =2\pi c\delta \lambda_{i}/\lambda_{0}^{2}$. We thus arrive at:

(7) $$w_{i}\;\approx \frac{N_{A}\left(1-\sqrt{1-S_{i}}\right)P\lambda_{0}}{\pi c}\frac{\Delta \lambda_{i}}{\delta \lambda_{i}}$$

As can be seen, the molecular information is encoded in the second factor, Δλ_*i*_/δλ_*i*_, while the prefactor accounts for the sensor configuration.

We now make two approximations. First, the coupling efficiency is only known on average, i.e., $\bar{S}$, and so we take $S_{i}\approx \bar{S}$. Secondly, while the linewidth δλ_*i*_ is known for all *i*, its fluctuations are negligible (δλ_*i*_ < 3σ) when compared with those for Δλ_*i*_. This motivates taking $\delta \lambda_{i}\;\approx \bar{\delta \lambda }$, where $\bar{\delta \lambda }$ is the average linewidth of the trace containing the molecular events under analysis. Therefore, Equation (7) can be approximated by following a map from Δλ_*i*_ to *w_i_*:

(8)

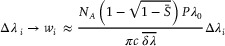




Equation (8) is applicable to any PE‐WGM experiment. Here we are interested in the work associated with the emergence of each step‐like event. Using Equations (3) and (8), this is given by:

(9) $$w_{0\to 1}=\frac{\;\lambda_{0}PN_{A}\left(1-\sqrt{1-\bar{S}}\right)A}{\pi c\bar{\delta \lambda }}\;$$

where state 0 refers to the conformation associated with the baseline, while state 1 refers to the conformation adopted for the duration of a step‐like event of amplitude *A*.

We can apply Jarzynski's estimator to calculate Gibbs free energy measured by the sensor.^[^ ^21^, ^22^, ^23^ ^]^ We find that we can measure the free energy with an unprecedented precision of 0.03 kJ mol^−1^ and up to a maximum of 2.25 kJ mol^−1^, on the same order of magnitude as the maximum Δ*G_c_* of 3PGK ≈ 4 kJ mol^−1^.^[^ ^25^ ^]^

Suppose we have μ step‐like signals. As per Equation (9), this leads to a set of work values *w*  =  (*w*_1_, *w*_2_, . . .,  *w*_μ_). Our goal is to use these to estimate the free energy difference Δ*G*_0 → 1_ using the Jarzynski equality:

(10) $$\exp\left(\beta \Delta G\right)=\langle \exp\left(\beta w\right)\rangle \;$$

where $\beta =\frac{1}{k_{B}T}\;,\;k_{B}$ – the Boltzmann constant, *T* – temperature,

(11) $$\langle \exp\left(\beta w\right)\;\rangle :=\;\int dw\;p\left(w|\Delta G\right)\exp\left(\beta w\right)$$

Rearranging, we see that

(12) $$\Delta G=\frac{1}{\beta }\;\ln\left[\int dw\;p\left(w|\Delta G\right)exp\left(\beta w\right)\right]$$

By applying Monte Carlo integration and error propagation to this equation, the following (asymptotic) estimator for ∆G can be constructed:

(13) $$\Delta G_{J}\pm \;\sigma_{\Delta G_{J}}=\frac{1}{\beta }\;\left\{\ln\left[m\left(w\right)\right]\pm \frac{1}{\sqrt{\mu }}\frac{\sigma \left(w\right)}{m\left(w\right)}\right\}$$

where

(14) $$m\;\left(w\right)=\frac{1}{\mu }\;\sum_{i=j}^{\mu }\exp\left(\beta w^{j}\right)$$

and

(15) $$\sigma {\left(w\right)}^{2}=m{\left(w\right)}^{2}+\frac{1}{\mu }\sum_{i=1}^{\mu }\exp\left(2\beta w^{j}\right)$$

This free energy penalty is plasmonic near‐field intensity‐dependent and must be overcome in order for turnover to be possible. The total free energy change of the system, Δ*G_system_*, is therefore the sum of Δ*G_C_* and Δ*G_P_*:

(16) $$\Delta G_{system}=\Delta G_{C}+\Delta G_{p}^{T}$$

As Δ*G_system_* must be negative for enzyme closing/opening to be thermodynamically favorable (where Δ*G_C_* is negative and $\Delta G_{p}^{T}$ is positive), enzyme turnover can theoretically be halted if $\Delta G_{p}^{T}$ is large enough to cause Δ*G_system_* to be positive, leading to the hypothesis that enzyme reactions can be controlled by PE‐WGM biosensors, as discussed in Houghton et al. (2024).^[^ ^7^ ^]^ We observe deviation from linearity at higher WGM intensity (*I* > 500 MW cm^−2^), suggesting that enzyme motion starts to be affected (Figure 3b). However, as the maximum $\Delta G_{p}^{T}$ values do not exceed Δ*G_c_*, there is no perturbation of enzyme turnover. Analysis of rate constant variation with hotspot intensity (Figures S2 and S3, Supporting Information) suggests that some influence is exerted on the enzyme, as shown by increasing *k* ^Δ*t*^(3PGK) versus *I*, while $k_{1}^{\Delta t}$(Adk) decreases. These “premature” influences on the kinetics could be due to the near‐field modifying dipoles within the enzyme involved with conformational change, substrate binding, or catalysis.^[^ ^7^, ^42^, ^43^, ^44^ ^]^ If these kinetic influences are indeed occurring, they appear to be applied to the enzyme in a direction‐ and enzyme‐specific manner.

We further hypothesized that the work done on 3PGK and Adk would differ substantially due to the difference in volume of the plasmonic hotspot that the two enzymes occupy.^[^ ^7^, ^11^, ^45^ ^]^ Consistent with this, the plots superimpose when enzyme diameter, a 1D measure of enzyme size, is taken into account (Figure 3c). Taken together, these observations could be considered to reflect and quantify a force exerted on a macromolecule by the optoplasmonic sensor, where the force is constant across enzymes. The electric field of the plasmonic hotspot can thus influence the movement of an immobilized enzyme by providing a resistive force against movement, akin to that seen in optical tweezers.^[^ ^46^ ^]^ Since the measured work on the enzyme is found to vary in a linear manner with volume/polarisability and applied PE‐WGM intensity, we introduce the effective force exerted by the enzyme without further calibration: we use the identity that effective force 𝐹 applied along the average diameter of the enzyme $\bar{d_{E}}$ is equal to the measured work.

(17) $$F=T_{S}\;I$$

where *T_S_* is trap stiffness (see Experimental Section). We find *T_S_* to be on the same order of magnitude across measurements with enzymes of different molecular weights and exhibiting different conformational motions (**Table** **1**). We can therefore assume, to the first approximation, that the sensor behaves similarly to an optical tweezer with forces proportional to polarisability (volume) of the dielectric particle, here an active enzyme, and the applied light intensity.

**Table 1:** Table of conversion from *w* as a function of *I* change to trap stiffness (*T_S_*).

Analyte	***Δw***/***ΔI*** [J cm^2^ MW^−1 ^mol^−1^]	$\bar{d_{E\;}}$ [nm]^a)^	***T***_***S***_ [nN cm^2^ W^−1^]
3PGK (reverse)	2.25 ± 0.132	4.65^b)^	0.802
Adk (forward)	1.75 ± 0.0485	3.76^c)^	0.774
Adk (reverse)	1.67 ± 0.0843	3.76^c)^	0.737

^a)^ Volume as predicted by ProteinVolume 1.3;^[^ ^47^ ^]^

^b)^ *Geobacillus stearothermophilus* (PDB entry 1PHP)^[^ ^48^ ^]^ = 5.28×10^−26 ^m^3^;

^c)^ *Aquifex aeolicus* (PDB entry 2RH5)^[^ ^49^ ^]^ = 2.78×10^−26^ m^3^

## 3 Discussion

Here we introduce an exceptionally sensitive approach for quantifying the thermodynamic penalties imposed on analyte molecules such as enzymes by a PE‐WGM sensor during measurements, previously overlooked in the context of optoplasmonic WGM single molecule studies. This research marks a progression in biochemical optoplasmonic analysis by quantifying the subtle, yet inherent, work done by the sensor while detecting enzyme conformational change. The exquisite sensitivity of the WGM method permits measurement of the minuscule forces exerted by active enzymes and expedites assessment of the influence of the probing light field on enzyme activity through quantification of the work done by the enzyme in moving against optical field gradients of the sensor. Our work therefore provides a quantitative tool for WGM biosensor researchers who aim to optimize measurement accuracy and reliability through understanding and quantification of the perturbation imposed by the sensor. Our work also provides a potential means to control biomolecules and enzyme activity with the spatial and temporal precision of a WGM‐based optoplasmonic sensor.^[^ ^7^ ^]^ It should be noted that the conformational changes observed here are classed as Tier 0 protein motions – large scale conformational changes such as domain motions, as defined by Ansari et al. (1985),^[^ ^50^, ^51^ ^]^ and that these are apparently unaffected by the optoplasmonic sensor in this study. However, thermodynamic penalties may also apply to faster Tier 1 and Tier 2 conformational changes such as loop fluctuations and amino acid side‐chain rotations that are also relevant for catalysis. These are outside the time resolution of our sensor and hence do not give rise to direct sensor signals but their Δ*G_c_* values are small enough to be affected by the optoplasmonic sensor. These Tier 1 and Tier 2 motions could be affected by the optoplasmonic forces imposed on the enzymes in this study, and this would help to explain the observed changes in rate constants (Figures S2 and S3, Supporting Information).

Control of enzyme activity at the single molecule level by a probing light source is of substantial interest, especially where other methods of control are destructive or unfeasible. For example, photoswitchable inhibitors are often used to selectively control enzyme activity by illumination, converting an inhibitor from inactive to active, but these are not available for all enzymes.^[^ ^52^ ^]^ Other methods of activity control such as altering pH or temperature are blunt instruments that are often irreversibly deleterious. Optoplasmonic WGM technology would benefit from being applicable as a reversible controller of activity, allowing switch‐like control, and from being applicable directly to the enzyme, similar to optical tweezers, but without the possible bias that may affect native movements during the data acquisition phase.^[^ ^53^, ^54^ ^]^ Particularly interesting uses of optoplasmonic WGM include error‐free *de novo* DNA synthesis and control of membrane‐bound proteins; optical tweezer investigations of membrane proteins, for example, have been limited to protein folding investigations due to the difficulty in attaching DNA handles to membrane‐bound proteins.^[^ ^55^, ^56^ ^]^

This investigation uses a highly sensitive optoplasmonic WGM sensor, as employed in several previous biomolecular studies.^[^ ^11^, ^12^, ^57^ ^]^ Plasmonic nanorods, used here, however, have a limited enhancement effect on field intensity, reducing the probability of reaching a near‐field strength sufficient to substantially impact enzyme activity. Higher input power is also stymied by an optothermal effect known as thermal broadening and narrowing whereby the transmission spectrum of the silica microsphere at high input power appears triangular in shape during up‐scanning, rather than Lorentzian, with a sharp dip during down‐scanning processes;^[^ ^58^ ^]^ this could potentially be circumvented by changes in buffer solution composition.^[^ ^59^ ^]^ Limitations also extend to the use of Jarzynski's equality, used here to estimate free energy changes from non‐equilibrium work done values, which can be biased in finite datasets.^[^ ^30^, ^31^, ^32^ ^]^ This bias decreases as sample size increases, so should be rendered inconsequential by the fact that we report the results of 6603 data points from 43 datasets. Variability in this investigation could have arisen from several sources, including the estimation of the values of near‐field enhancement and hotspot intensity exerted on the molecules; these estimations rely on enzymes binding to the ideal location on the plasmonic nanorods. Thermorefractive noise, moreover, can cause near‐field enhancement and hotspot intensity to change over the course of the experiment.^[^ ^7^ ^]^ Variability may also arise from perturbation of enzyme properties: deviation from linearity in *w* versus *I*, for example, could result from changes in the folding landscape and in Tier 1/2 conformational motions, as mentioned above.

Improvements in PE‐WGM, including the use of different shapes of plasmonic enhancer to increase the near‐field gradient, plus further understanding of polarisability changes in aqueous solution,^[^ ^60^ ^]^ could facilitate the breakthroughs required to create a setup capable of manipulating enzyme turnover in a controllable manner.^[^ ^7^, ^61^ ^]^ Additionally, other methods of control could be combined with optoplasmonic force, including laser heating,^[^ ^62^ ^]^ plasmonic heating,^[^ ^7^ ^]^ and microfluidic segmented flow strategies.^[^ ^63^ ^]^ Combining these methods in a lab‐on‐a‐chip format could provide exceptional enzyme reaction control capabilities at single molecule level, allowing for greater intensity manipulation by addition of waveguides to excite WGMs, for example for improvements to existing biosynthetic applications and also for the development of novel biosynthetic applications.

## 4 Experimental Section

### Sample Preparation

3PGK from *Geobacillus stearothermophilus* was cloned with a hexahistidine tag at both the N‐ and C‐termini. Adk from *Aquifex aeolicus* was cloned with a C‐terminal hexahistidine tag and Cys residue (HHHHHHC*). DNA encoding these proteins was prepared and inserted into the pET30a(+) plasmid by GenScript. 3PGK and Adk were expressed using BL21(DE3) cells (NEB) incubated in LB medium with 50 µg mL^−1^ kanamycin. At OD_600_ 0.6‐0.8, 400 µm IPTG was added, and incubation was continued at 20 °C for 18 h. Cells were harvested by centrifugation at 4 °C at 4000 g for 30 min, resuspended in 5 mL HisA buffer (50 mm Tris‐HCl, 300 mm NaCl, 20 mm imidazole, pH 7.5), and sonicated at 60% amplitude for 10 s pulses (10 s rest) for 5–10 min on ice. The soluble fraction was separated by centrifugation at 4 °C for 30 min at 26 000 g.

Protein was purified from the soluble fraction using an ÄKTA pure (Cytiva) system by immobilized metal affinity chromatography (IMAC) with HisTrapFF (Cytiva) columns. A gradient of imidazole was used to separate the protein over 15 min from 20 to 500 mm imidazole. Aliquots containing Adk were pooled and concentrated using a Vivaspin 20 3000 MWCO polyethersulfone centrifugal concentrator (Sartorious VS2092) at 4 °C and 3000 g until sample volume was 5 mL. A second purification step, size exclusion chromatography (SEC) with a HiLoad 16/600 Superdex column with 50 mm HEPES pH 7.5, 150 mm NaCl, was then performed. 2 mL fractions were collected over 1.5 column volumes and fractions containing the enzyme were concentrated again to ≈10 mg mL^−1^.

### Plasmonically Enhanced Whispering Gallery Mode Setup

The setup is depicted in Figure 1a. A Toptica DL Pro 760–815 nm tunable CW optical fiber coupled laser was connected to an attenuator via a polarisation maintaining fiber. A collimator and optics focus the beam onto the back of an NSF11 quartz prism at ≈6°. The reflected beam off this boundary was focussed onto a photodetector and spectrum collected. Additional details can be found in Subramanian et al. (2021)^[^ ^12^ ^]^ and Toropov et al. (2023).^[^ ^57^ ^]^ No PDH‐lock was used in this data acquisition setup. Monitoring of the spectrum was performed using a custom LabView data acquisition program with a feedforward factor of −0.4.^[^ ^64^ ^]^ The WGM microcavity was coupled to the evanescent field formed at this boundary where the beam was reflected, hung from a custom‐built mount, and positioned using an XYZ translational stage.

The WGM microcavities used here were spherical microresonators. These were fabricated on a home‐built setup at the tip of an SMF‐28e single‐mode optical fiber (Corning) to a radius of 40–45 µm with a 30 W CO_2_‐laser (Synrad 48‐2) at 10–16% maximum power.

The 300 µL poly‐dimethylsiloxane sample chamber was filled with 0.02 m HCl and spherical microresonator coupled to the beam. 2 µL of plasmonic gold nanorods (AuNRs), with localized surface plasmon resonance of 780 nm and cetrimonium bromide capping (Nanopartz A12‐10‐780‐CTAB), were added to the chamber until 5–8 were bound, monitored at 50 Hz scanning rate and identified by rapid wavelength and full‐width‐at‐half‐maximum (FWHM) shifts. The sample chamber was rapidly emptied and pH neutralized to sequester any additional AuNRs from binding by washing with 50 mm HEPES, pH 7.5. HEPES was used throughout this investigation due to its limited interactions with the PE‐WGM sensor, preventing additional noise.

### Enzyme Binding to Gold Nanorods

To bind 3PGK, the surface chemistry of the AuNRs was modified by incubating the microsphere‐AuNRs for 20 min at room temperature in 1.67 µm dithiobis(C2‐NTA) (Dojindo D550), 15 µm thiol‐dPEG4‐acid (Sigma‐Aldrich QBD10247) and 8.33 µm TCEP‐HCl (tris(2‐carboxyethyl)phosphine‐HCl) in 50 mm citrate‐1 m NaCl, pH 3.1. The chamber was again washed with 50 mm HEPES, pH 7.5, and resultant plasmonic constructs then charged with nickel ions by incubation for 2 min in 0.1 m NiSO₄. The chamber was again washed with 50 mm HEPES, pH 7.5, before addition of 3PGK to a final concentration of 100–200 nm.

Adk was bound to AuNRs on the surface of the microresonator by formation of gold‐thiol bonds involving the C‐terminal Cys residue. 5–8 AuNRs were bound to the microsphere, as above, before washing with 50 mm HEPES, pH 7.5. The microsphere‐AuNRs were immersed in 25 mm TCEP‐50 mm HEPES, pH 7.5, to ensure all Adk is in a monomeric form and that sulfhydryl groups available for interaction with the gold surface. WGM wavelength was monitored: Adk (at a final concentration of 100–200 nm) was observed binding to the surface of gold nanorods.

### Enzyme Turnover and Signal Identification

Observation of 3PGK turnover in the reverse direction occurred in the presence of 50–200 µm 3‐phosphoglycerate and 6–18 µm ATP in 50 mm HEPES, pH 7.5. These concentrations were chosen such that any changes in substrate concentration over the course of an experiment were minimal, ensuring unidirectionality and reducing effects on kinetics/thermodynamics, and also minimizing additional noise that could be caused by too high a concentration of solute on the sensor. Signals were taken as wavelength shifts greater than three times the standard deviation (3σ) of the WGM resonance wavelength change, either as spikes or double peaks, with no corresponding significant FWHM change. A custom MATLAB analysis program was used to identify and record the amplitude of signals.^[^ ^65^ ^]^ On average, 154 signals were obtained per measurement.

Adk turnover was observed in both forward and reverse directions. Forward turnover was performed with 100–200 µm ATP and 50–100 µm AMP in 200 mM MgCl_2_‐50 mm HEPES, pH 7.5. Reverse turnover was performed in 200 mm MgCl_2_‐50 mm HEPES, pH 7.5 with 1–100 µm ADP substrate. Turnover signals again appeared as wavelength shifts above 3σ with no significant corresponding FWHM changes, generally as spike‐like signals. On average, 176 signals were obtained per measurement. The signal amplitudes were identified and recorded with the same MATLAB analysis package as above, taking into account slow temperature fluctuations using a first‐order Savitzky–Golay filter (Supporting Information in Subramanian et al., 2021).^[^ ^12^ ^]^

### Calculating Thermodynamic Quantities

Using the values of signal amplitude collected from PE‐WGM experiments above, where the enzyme converts from state 0 (open) to state 1 (closed), the work done on the enzyme by the sensor, *w* (kJ/mol) per signal is calculated as:

(9) $$w_{0\to 1}=\frac{\;\lambda_{0}PN_{A}\left(1-\sqrt{1-\bar{S}}\right)A}{\pi c\bar{\delta \lambda }}\;$$

where wavelength, λ_0_, input laser power, *P*, average coupling percentage, $\bar{S}$, signal amplitude, *A*, speed of light, *c*, mean FWHM, $\bar{\delta \lambda }$, and Avogadro's Number, *N_A_*, had intially. These were used to generate empirical probability distributions of work done on an enzyme during turnover as observed by PE‐WGM at a given WGM intensity.

μ number of step‐like signals were used to estimate the free energy difference Δ*G*_0 → 1_ using Jarzynski's estimator:

(13) $$\Delta G\pm \;\sigma_{\Delta G}=\frac{1}{\beta }\;\left\{\ln\left[m\left(w\right)\right]\pm \frac{1}{\sqrt{\mu }}\frac{\sigma \left(w\right)}{m\left(w\right)}\right\}$$

where

(14) $$m\;\left(w\right)=\frac{1}{\mu }\;\sum_{i=j}^{\mu }\exp\left(\beta w^{j}\right)$$

and,

(15) $$\sigma {\left(w\right)}^{2}=m{\left(w\right)}^{2}+\frac{1}{\mu }\sum_{i=1}^{\mu }\exp\left(2\beta w^{j}\right)$$

and $\beta =\frac{1}{k_{B}T}\;.$ These equations show how the values of *w* can be used to calculate Gibbs free energy changes (Δ*G* – in kJ/mol) when an enzyme undergoes large scale conformational change during turnover, while additionally considering the Boltzmann constant, *k_B_* (m^2^ kg s^−2^ K^−1^), temperature, *T* (K) and number of signals, μ.

### Calculating Intensity at the Enzyme

The WGM toolkit by Balac et al. (2019)^[^ ^38^ ^]^ was used to calculate the field distribution of a WGM. The effective mode volume,  *V_eff_*, is calculated using:

(22) $$V_{\mathrm{eff}}=\frac{\int \varepsilon \left(r\right){\left|E\left(r\right)\right|}^{2}dr}{\Lambda \varepsilon \left(r_{0}\right){\left|E\left(r_{0}\right)\right|}^{2}}\;$$

where ε(r) is the spatial distribution of the relative permittivity, E(r) is the numerical field distribution, Λ is the enhancement factor due to the localized surface plasmon resonance of the AuNR, and r_0_ is the position of this AuNR. The intracavity photon number,  *N_in_*, is calculated using $N_{\mathrm{in}}=\frac{\kappa_{\mathrm{in}}}{\kappa^{2}h\omega }\;P$, where κ_in_ is the coupling rate and κ is the total loss rate of the microsphere, *h* is Planck's constant, ω  = 2π*c*/λ where c is the speed of light. This allows the intensity *at the enzyme*, *I*, to be calculated as *I*  = *h*ω*N*_in_ *c*/*V*_eff_ .^[^ ^57^ ^]^

### Optoplasmonic Force/Work Equation and Trap Stiffness

The trap stiffness *T_S_*, a quality of the sensor that describes the force exerted on the enzymes per WGM intensity (N cm^2^ W^–1^), is defined as:

(23) $$T_{S}=\frac{\left(\Delta w/\Delta I\right)}{\bar{d_{E\;}}N_{A}}$$

with Δw/ΔI the work‐done proportionality constant extracted from Figure 2a, $\bar{d_{E}}$ the average enzyme diameter, and *N_A_* Avogadro's number. The trap stiffness was found to be on the same order of magnitude in measurements with enzymes of different molecular weights and exhibiting different conformational motions. Because of this, to a first approximation, this study assumed that the sensor behaved similarly to an optical tweezer with forces proportional to polarisability (volume) of the dielectric particle and the applied light intensity:

(24) $$F=T_{S}\;I$$

The average force experienced is calculated using $w=F\;\bar{d_{E}}\;N_{A}$, expressing force exerted on a single enzyme as:

(25) $$F=\frac{\left(\Delta w/\Delta I\;I\right)}{\bar{d_{E}}\;N_{A}}$$

where *F* is force (N).

## Conflict of Interest

The authors declare no conflict of interest.

## Supporting information

Supporting Information

## Data Availability

The data that support the findings of this study are available in the supplementary material of this article.
