# Supplementary material for: Single Molecule Thermodynamic Penalties Applied to Enzymes by Whispering Gallery Mode Biosensors
Source: Adv Sci (Weinh). 2024 Jul 12;11(35):2403195. doi: 10.1002/advs.202403195 (PMC11425209; doi:10.1002/advs.202403195)
Supplement: Supplementary file 1 — Supporting Information [file ADVS-11-2403195-s001.pdf]

Supporting Information

Single Molecule Thermodynamic Penalties Applied to Enzymes by Whispering Gallery Mode Biosensors

Matthew C. Houghton[1,2,3,*], Nikita A. Toropov[1,2,4] , Deshui Yu[5] , Stefan Bagby[3] , Frank Vollmer[1,2,**]

* M.C.Houghton@exeter.ac.uk

** F.Vollmer@exeter.ac.uk

Binding and Turnover Kinetics

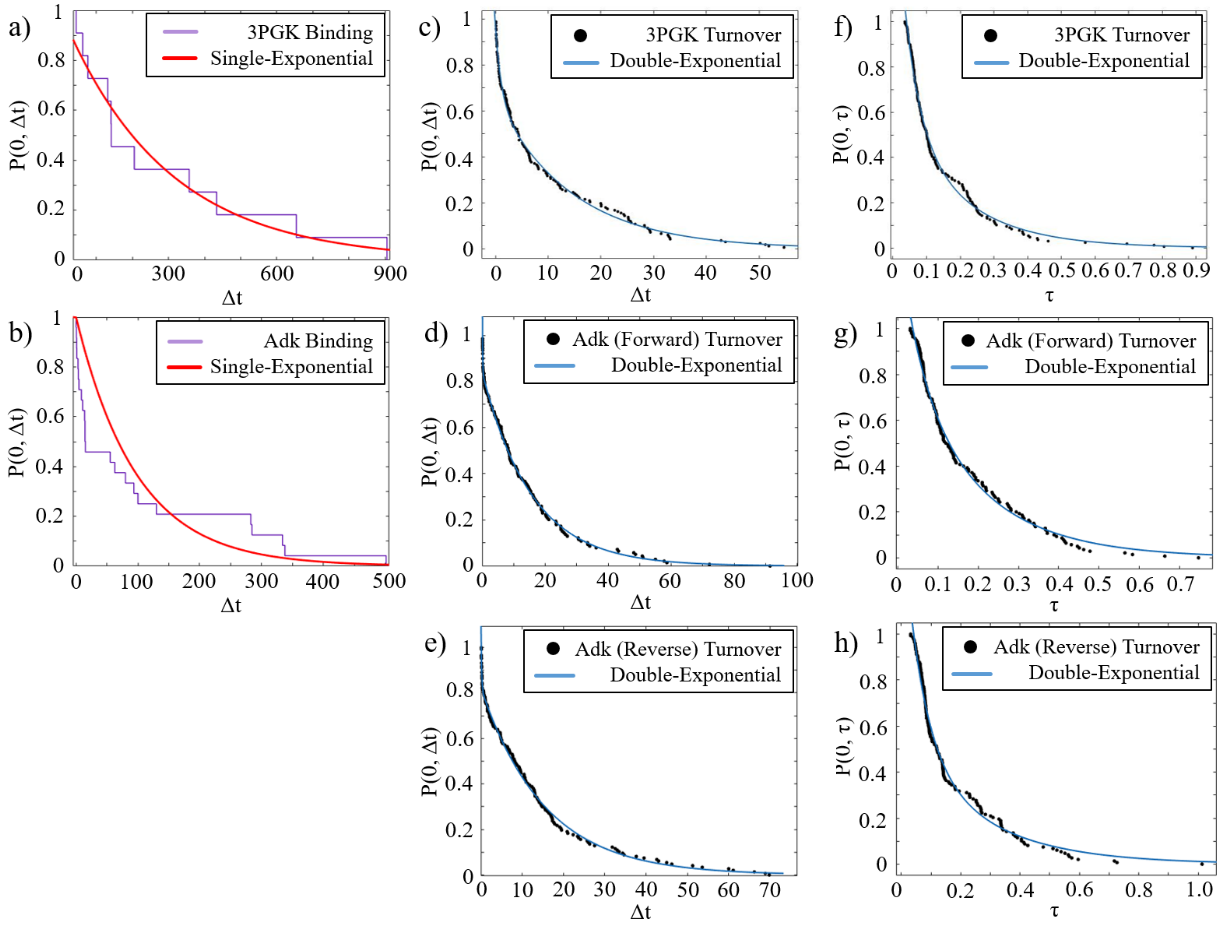

Figure S1. Survival plot of binding to the optoplasmonic sensor of **(a)** 3PGK and **(b)** Adk. This demonstrates single-molecule events due to fitting an exponential curve of $\boldsymbol{P(0,\Delta t) = e^{-R\Delta t}}$.[57, 66] Different kinetic parameters of enzyme turnover can be estimated by fitting $\boldsymbol{P(0,\Delta t) = \sum_{i=1}^{\leq 2} A_i\, e^{-k\Delta t}}$ or $\boldsymbol{P(0,\tau) = \sum_{i=1}^{\leq 2} A_i\, e^{-k\tau}}$.[12] These survival plots can be seen here as the wait time ($\boldsymbol{\Delta t}$) of **(c)** 3PGK (reverse), **(d)** Adk (forward), and **(e)** Adk (reverse). Survival plots of signal durations reveal values of $\boldsymbol{k}$ that tend towards $\boldsymbol{k_{cat}}$with **(f)** 3PGK (reverse: double-exponential), **(g)** Adk (forward: double-exponential), and **(h)** Adk (reverse: double-exponential).

Table S1. Bulk rate constants vs single-molecule rate constants where I < 150 MW cm^{-2}

Enzyme	k_{cat}	$k^{\Delta t}$	$k_1^{\Delta t}$	$k_2^{\Delta t}$	k^{τ}	k_1^{τ}	k_2^{τ}
**3PGK (R)**	9	0.0747	1.62	0.125	9.85	12.2	3.49
**Adk (F)**	-	0.0575	3.49	0.0504	11.2	15.8	6.74
**Adk (R)**	14.9	0.1076	1.81	0.154	8.08	18.0	3.12

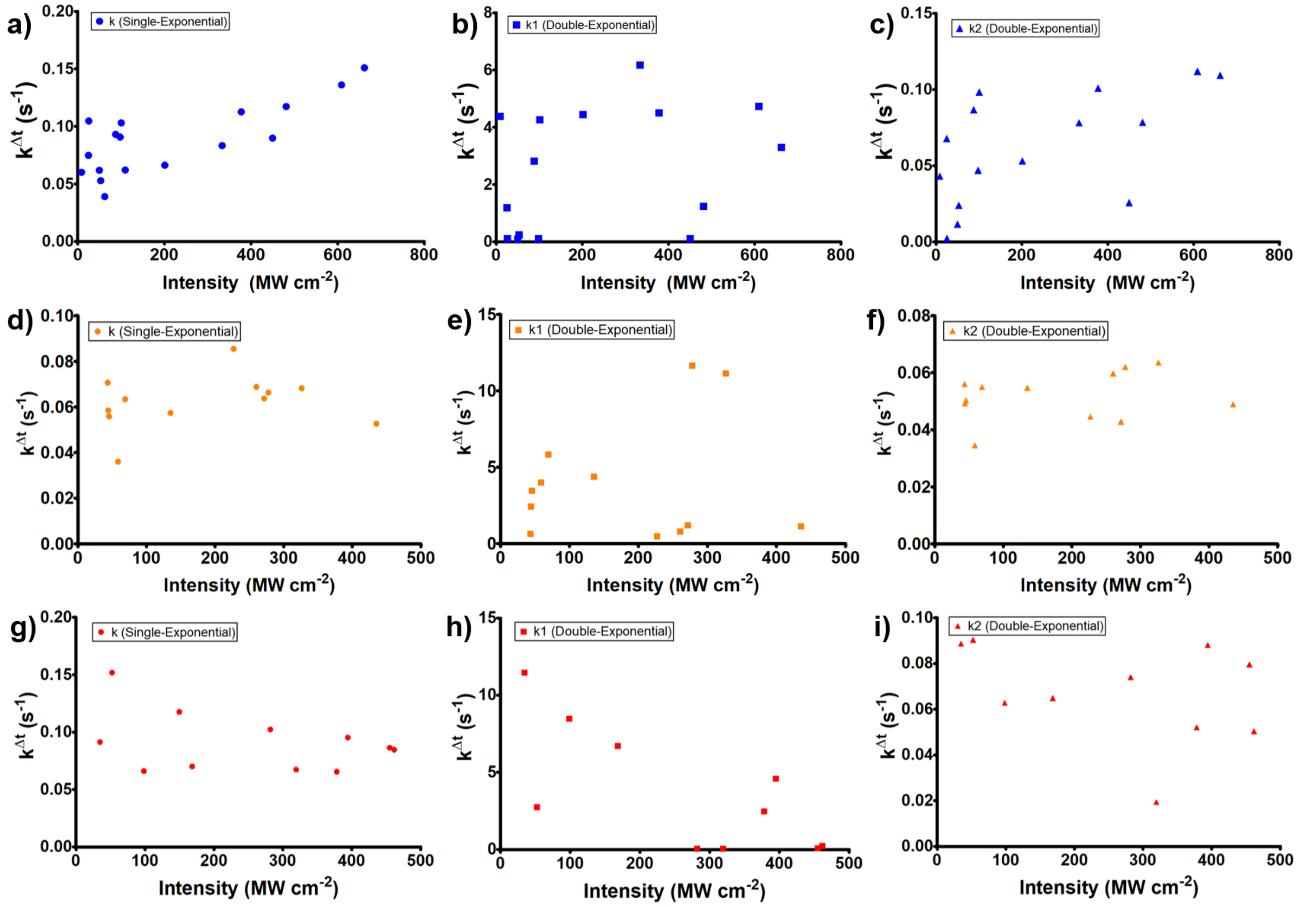

Figure S2. Kinetics of **(a-c)** 3PGK, **(d-f)** Adk forward, and **(g-i)** Adk reverse turnover in the $k^{\Delta t}$ regime. **(a, d, g)** depict the fitting of a single-exponential, **(b, e, h)** $k_1^{\Delta t}$ of a double exponential and **(c, f, i)** $k_2^{\Delta t}$ of a double exponential with increasing hotspot intensity.

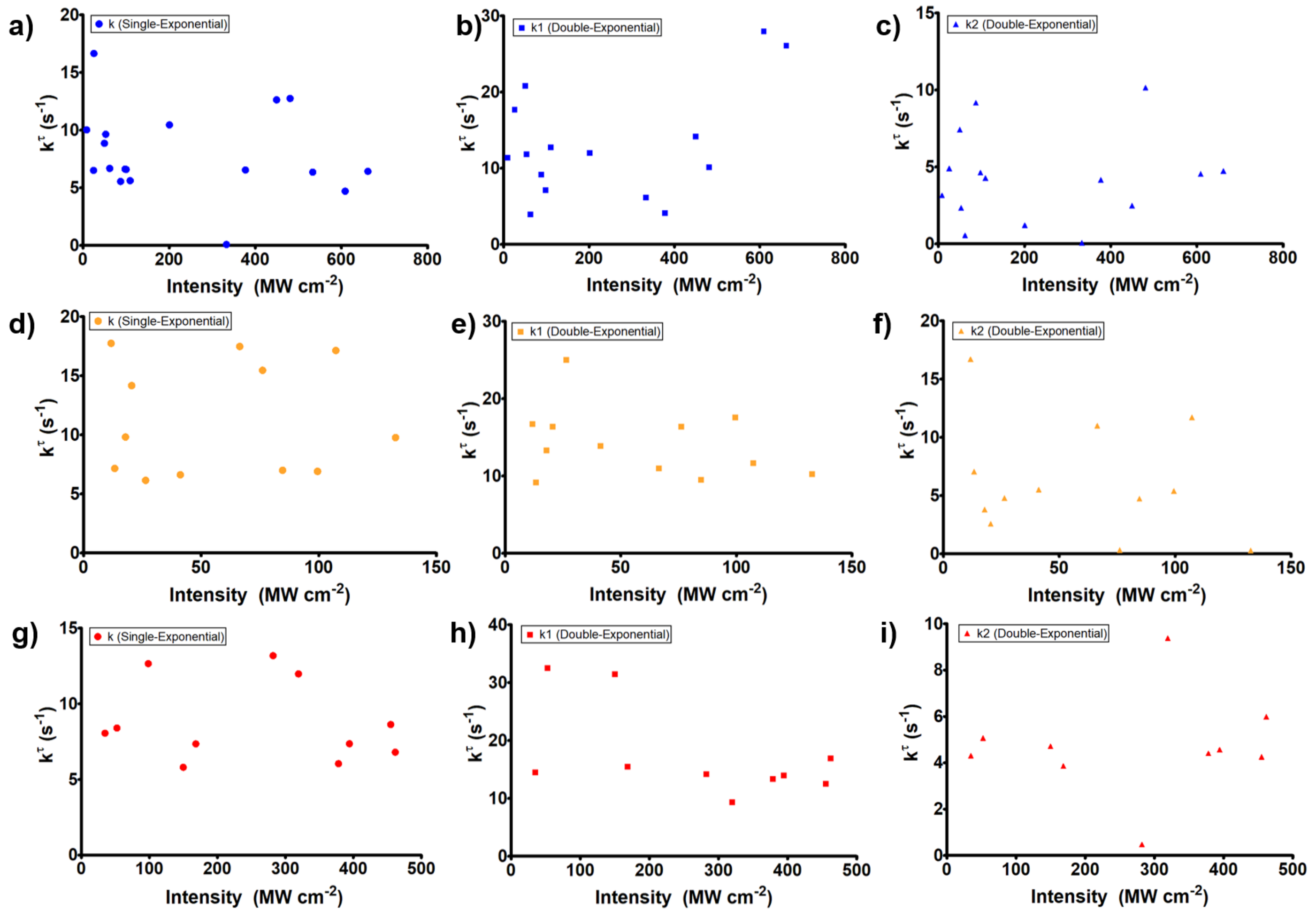

Figure S3. Kinetics of **(a-c)** 3PGK, **(d-f)** Adk forward, and **(g-i)** Adk reverse turnover in the k^{τ} regime. **(a, d, g)** depict the fitting of a single-exponential, **(b, e, h)** k_1^{τ} of a double exponential and **(c, f, i)** k_2^{τ} of a double exponential with increasing hotspot intensity.

Raw Thermodynamic and Force Data

Table S2. Data from optoplasmonic monitoring of 3PGK turnover.

**3PGK (Reverse)**						
**WGM Intensity [MW cm^{-2}]**	**Mean w [kJ/mol]**	**w Error**	**ΔG_P [kJ/mol]**	**ΔG_P Error**	**Predicted Force [pN]** [a)]	**Measured Force (pN)** [b)]
**8.53**	0.0331	0.0012	0.0332	0.0012	0.006845	0.011811
**25.3**	0.103	0.0059	0.1035	0.006	0.020302	0.036752
**52.79**	0.0783	0.0036	0.0785	0.0036	0.042362	0.027939
**62.12**	0.2006	0.0073	0.2011	0.0073	0.049849	0.071577
**97.72**	0.163	0.0064	0.1632	0.0063	0.078416	0.058161
**377.24**	1.0055	0.0221	1.0201	0.0224	0.302720	0.358778
**449.62**	0.8108	0.0498	0.8528	0.0729	0.360802	0.289306
**200.76**	0.3535	0.0175	0.3572	0.0188	0.161102	0.126134
**333.15**	0.7183	0.0283	0.7341	0.0329	0.267340	0.256301
**480.97**	0.8627	0.0328	0.8844	0.0387	0.385959	0.307825
**608.87**	1.2698	0.0576	1.3546	0.0726	0.488594	0.453085
**661.55**	1.3706	0.0577	1.4358	0.0577	0.530867	0.489052
**24.55**	0.0882	0.0016	0.0883	0.0016	0.019700	0.031471
**109.5**	0.1868	0.0028	0.187	0.0028	0.087869	0.066653
**87.35**	0.379	0.0079	0.3815	0.0078	0.070095	0.135233
**49.76**	0.1617	0.0053	0.1619	0.0053	0.039930	0.057697
**100.32**	0.2232	0.0038	0.2239	0.0038	0.080503	0.079641
**533.41**	0.9227	0.049	0.9333	0.0475	0.428040	0.329234
**662.16**	2.1585	0.0589	2.4111	0.1058	0.531357	0.770187

a) $\boldsymbol{F = T_S\, I}$

b) $\boldsymbol{F = \frac{w}{\overline{d_E}\, N_A}}$

Table S3. Data from optoplasmonic monitoring of Adk turnover.

WGM Intensity [MW cm^{-2}]	Mean w [kJ mol^{-1}]	w Error	ΔG_P [kJ mol^{-1}]	ΔG_P Error	Predicted Force [pN] [a)]	Measured Force [pN] [b)]
**Adk (Forward)**						
**271.445**	0.4787	0.0158	0.4846	0.0165	0.21001	0.21001
**68.81**	0.1157	0.0033	0.1161	0.0034	0.05324	0.05324
**260.16**	0.4839	0.0097	0.4892	0.0101	0.20128	0.20128
**226.93**	0.3735	0.009	0.3782	0.0099	0.17557	0.17557
**45.46**	0.0297	0.0144	0.0365	0.0143	0.03517	0.03517
**44.07**	0.0614	0.0019	0.0615	0.0019	0.03410	0.03410
**43.39**	0.0715	0.002	0.0716	0.0020	0.03357	0.03357
**134.96**	0.2525	0.005	0.2538	0.0050	0.10441	0.10441
**435.05**	0.8349	0.0219	0.8501	0.0245	0.33658	0.33658
**326.31**	0.5145	0.0174	0.5232	0.0178	0.25246	0.25246
**277.6**	0.4388	0.0122	0.4424	0.0119	0.21477	0.21477
**58.44**	0.0875	0.0032	0.0877	0.0032	0.04521	0.04521
**Adk (Reverse)**						
**WGM Intensity [MW cm^{-2}]**	**Mean w [kJ mol^{-1}]**	**w Error**	**ΔG_P [kJ mol^{-1}]**	**ΔG_P Error**	**Predicted Force [pN] [a)]**	**Measured Force [pN] [b)]**
**52.22**	0.0665	0.0034	0.0668	0.0034	0.03846	0.0293658
**149.83**	0.4152	0.0145	0.4200	0.0145	0.11036	0.1833487
**378.15**	0.5615	0.0201	0.5763	0.0213	0.27854	0.2479535
**168.3**	0.2172	0.0051	0.2185	0.0053	0.12397	0.0959136
**461.63**	0.8368	0.0359	0.9153	0.0705	0.34002	0.3695235
**394.23**	0.4735	0.0091	0.4766	0.0096	0.29038	0.2090934
**98.33**	0.1644	0.0056	0.1657	0.0059	0.07243	0.0725976
**281.91**	0.4768	0.0089	0.4805	0.0088	0.20765	0.2105507
**455.01**	0.7846	0.0173	0.7992	0.0202	0.33515	0.3464725
**38.22**	0.1171	0.0033	0.1175	0.0033	0.02550	0.0517103
**352.53**	0.615	0.0133	0.6208	0.0137	0.23518	0.2715786
**428.60**	0.6812	0.0127	0.6903	0.0145	0.28593	0.3008119

a) $\boldsymbol{F = T_S\ I}$
b) $\boldsymbol{F = \frac{w}{\bar{d_E}\ N_A}}$

References

[1] M. D. Baaske, F. Vollmer, Optical observation of single atomic ions interacting with plasmonic nanorods in aqueous solution. *Nature Photon* **10** 733-739 (2016). doi: 10.1038/nphoton.2016.177

[2] N. A. Toropov, M. C. Houghton, D. Yu, F. Vollmer, (Preprint) bioRxiv:2023.12.13.571444, submitted: 2023-12-14. doi: 10.1101/2023.12.13.571444

[3] S. Subramanian *et al.*, Sensing enzyme activation heat capacity at the single-molecule level using gold-nanorod-based optical whispering gallery modes. *ACS applied nano materials* **4** (5), 4576-4583 (2021). doi: 10.1021/acsanm.1c00176

[4] S. Frustaci, University of Exeter, Open Research Exeter (2023).
